# A novel loop-structure-based bispecific CAR that targets CD19 and CD22 with enhanced therapeutic efficacy against B-cell malignancies

**DOI:** 10.1093/procel/pwae034

**Published:** 2024-06-01

**Authors:** Lijun Zhao, Shuhong Li, Xiaoyi Wei, Xuexiu Qi, Qiaoru Guo, Licai Shi, Ji-Shuai Zhang, Jun Li, Ze-Lin Liu, Zhi Guo, Hongyu Zhang, Jia Feng, Yuanyuan Shi, Suping Zhang, Yu J Cao

**Affiliations:** State Key Laboratory of Chemical Oncogenomics, Shenzhen Key Laboratory of Chemical Genomics, Peking University Shenzhen Graduate School, Shenzhen 518055, China; State Key Laboratory of Chemical Oncogenomics, Shenzhen Key Laboratory of Chemical Genomics, Peking University Shenzhen Graduate School, Shenzhen 518055, China; State Key Laboratory of Chemical Oncogenomics, Shenzhen Key Laboratory of Chemical Genomics, Peking University Shenzhen Graduate School, Shenzhen 518055, China; State Key Laboratory of Chemical Oncogenomics, Shenzhen Key Laboratory of Chemical Genomics, Peking University Shenzhen Graduate School, Shenzhen 518055, China; State Key Laboratory of Chemical Oncogenomics, Shenzhen Key Laboratory of Chemical Genomics, Peking University Shenzhen Graduate School, Shenzhen 518055, China; State Key Laboratory of Chemical Oncogenomics, Shenzhen Key Laboratory of Chemical Genomics, Peking University Shenzhen Graduate School, Shenzhen 518055, China; The Shenzhen Pregene Biopharma Company, Ltd., Shenzhen 518118, China; Fundamenta Therapeutics Co., Ltd, Suzhou 215200, China; Department of Hematology, Huazhong University of Science and Technology Union Shenzhen Hospital, Nanshan Hospital, Shenzhen 518052, China; Department of Hematology, Huazhong University of Science and Technology Union Shenzhen Hospital, Nanshan Hospital, Shenzhen 518052, China; Department of Hematology, Peking University Shenzhen Hospital, Shenzhen 518036, China; Department of Hematology, Peking University Shenzhen Hospital, Shenzhen 518036, China; Shenzhen Cell Valley Biomedical Co., LTD, Shenzhen 518118, China; Shenzhen Key Laboratory of Precision Medicine for Hematological Malignancies, Base for International Science and Technology Cooperation: Carson Cancer Stem Cell Vaccines R&D Center, International Cancer Center, Shenzhen University Medical School, Shenzhen 518055, China; State Key Laboratory of Chemical Oncogenomics, Shenzhen Key Laboratory of Chemical Genomics, Peking University Shenzhen Graduate School, Shenzhen 518055, China; Institute of Chemical Biology, Shenzhen Bay Laboratory, Shenzhen 518132, China

**Dear Editor,**

Chimeric antigen receptor T (CAR-T) cells have achieved substantial advances in the treatment of B-cell malignancies. Despite high initial response rates, some patients relapse, characterized by targeted antigen loss (Shalabi et al., 2018). To overcome antigen escape and heterogeneity of antigen expression, CAR constructs that recognize dual or multiple antigens have been designed in various formats (Qin et al., 2018; Schneider et al., 2021; Zhao and Cao, 2019), thereby reducing the recurrence rate. The distance between T cells and target cells was identified as the key element in triggering robust CAR-T-cell activity. However, nearly all CD19/CD22-bispecific CARs reported in recent years have the problem of insufficient CD22 targeting (Qin et al., 2018), which is caused by the suboptimal spatial configuration of CD19 and CD22 antibodies. Given their small size, nanobodies have been included in our research on targeting distance optimization (Han et al., 2021; Mo et al., 2021). On this basis, optimal dual-targeting CARs with the desired functionalities were created by addressing various challenges, such as linker selection and antibody orientation.

To obtain the optimal CAR structure, using the G_4_S linker, hinge lama linker (De Munter et al., 2018), or β-stranded linker (Wang et al., 2013), we generated CD19/CD22 CARs in three different formats (Figs. S1 and S3A), denoted TanCAR-1, TanCAR-2, and LoopCAR-1, respectively. Although the bispecific CAR-T cells exhibited comparable cytotoxicity against CD19^+^ and/or CD22^+^ target cells (Fig. S3B), LoopCAR-1 demonstrated significantly increased cytokine production and enhanced proliferation (Fig. S3C and S3D). We collected primary B-ALL patient samples with distinct levels of CD19 and CD22 expression (Fig. S2). LoopCAR-1 exhibited enhanced cytotoxicity against B-ALL patient cells (Fig. S4A and S4B). Therefore, the loop structure could be an optimal design for a bispecific CD19/CD22 CAR and was selected for further functional analysis.

Another loop-type CD19/CD22 CAR designed with M971 and FMC63 scFv (designated as LoopCAR-4) demonstrated impressive activity in a phase I clinical trial (Spiegel et al., 2021). To investigate the key elements for optimal loop CAR designs, we further designed two additional CD19/CD22 loop CARs by replacing the linker and CD22 nanoantibody (Fig. 1A). We verified that, in contrast to the membrane-proximal binding position of M971 within the Ig-like domain of CD22 (d6–d7) (Ereño-Orbea et al., 2021), Nb25 potentially targeted an intermediate domain (d4) on CD22 (Figs. 1B and S5A–C). We then evaluated immunological synapse (IS) formation and observed greater PKC-θ enrichment in LoopCAR-1 cells than in CD22^+^ Nalm6-KO19 cells (Fig. 1C and 1D). The differences in the binding ability of LoopCARs to CD22 antigen are also consistent with the above conclusions (Fig. S6A), indicating that LoopCAR-1 may have a greater advantage in CD22 targets. Compared with the other two candidates, LoopCAR-1 and LoopCAR-2 had markedly greater effects on Nalm6 and Nalm6-KO19 cells (Figs. 1E and S6A). Interestingly, compared with LoopCAR-4, which employs a short G_4_S linker, LoopCAR-3, which is designed with a β-stranded linker and both utilizes M971, demonstrated enhanced CD22-redirected cytotoxicity. Furthermore, LoopCAR-1-treated Nalm6 and Nalm6-KO19 cells exhibited notable increases in T-cell activation and degranulation (Fig. 1F and 1G). Moreover, we observed more pronounced CAR-CD3ζ phosphorylation for LoopCAR-1 stimulated with Nalm6-KO19 cells compared to other loop CARs (Figs. 1H and S6B). After two rounds of challenge, all the CARs exhibited comparable cytotoxicity against CD19^+^ Nalm6-KO22 cells, whereas LoopCAR-1 had more persistent cell-killing activity than the other bispecific CARs (Fig. 1I). Additionally, we observed an increased population of effector T cells after restimulation in LoopCAR-1 (Fig. S6C) but a reduced expression of exhaustion markers (Fig. 1J), demonstrating the sustained antitumor activity of LoopCAR-1 in response to target cells experiencing CD19 antigen loss. Taken together, these results underscore the importance of antibody and linker selection in CAR design and indicate that LoopCAR-1 may represent an optimal bispecific CAR structure for target cells with various antigen densities.

**Figure 1.**
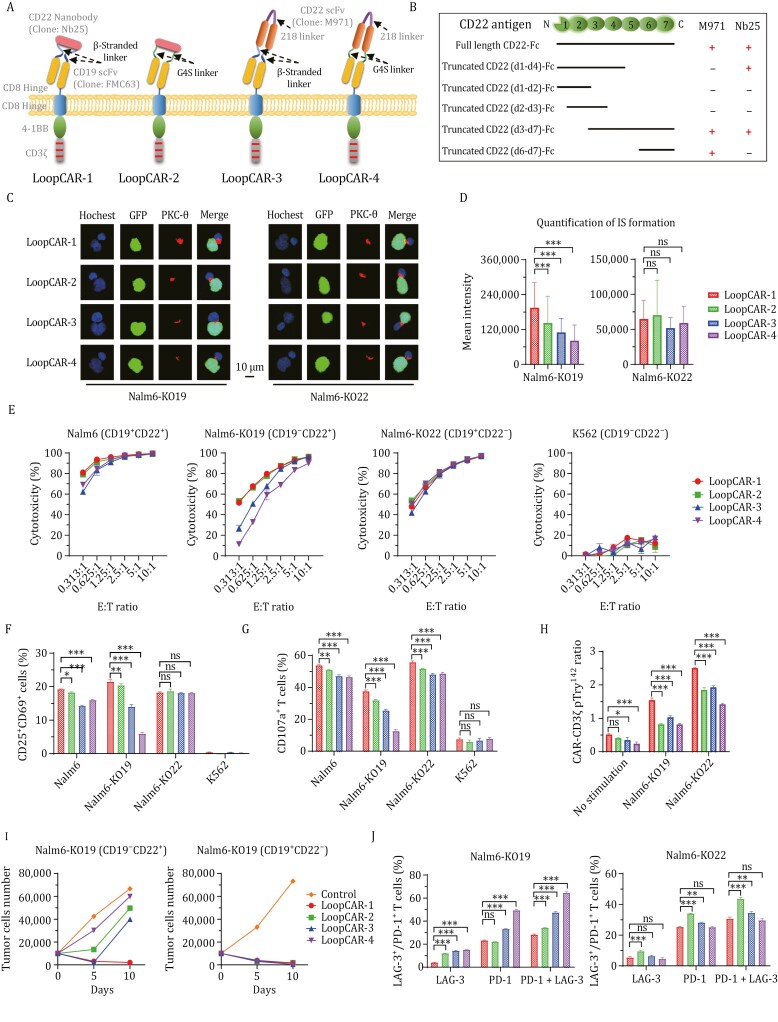
LoopCAR-1 exhibited an optimal loop CAR design. (A) Schematic structures of four loop-structure CARs. There were differences in the linker and CD22 binding domain. LoopCAR-4 is composed of a targeting domain generated by incorporating M971 scFv between the VL and VH regions of FMC63 scFv via the G_4_S linker, followed by the same hinge region, transmembrane domain, costimulatory domain, and CD3ζ activation domain as LoopCAR-1. (B) Schematic representation of the binding sites of two CD22 antibodies, M971 and Nb25, to the CD22 antigen. The symbol “+” indicates binding, and “−” indicates no binding. Nb25 recognizes the CD22 antigen d4 domain, whereas M971 recognizes the CD22 antigen d6–d7 domain. (C) Confocal representative synapse images from three independent experiments. Tumor cells (transduced with GFP) were cocultured with CAR-T cells for 1 h, and cell–cell conjugates were imaged at 100× oil objective magnification under a laser scanning confocal microscope (Nikon, A1R). Hoechst, anti-PKC-θ, GFP and an overlay of all the stains are shown. Scale bar = 10 μm. (D) Data on the accumulation of PKC-θ molecules at the IS between tumors and CAR-T cells. The graphic shows the means ± SDs of the PKC-θ fluorescence intensities for more than 20 cell–cell conjugates from three independent experiments. Asterisks indicate statistical significance using the Newman-Keuls multiple comparison test. *P < 0.05, **P < 0.01, ***P < 0.001, and ns: not statistically significant (≥0.05). (E) Cytotoxicity comparison of the four loop-structure CARs against the indicated tumor cells after 24 h of incubation at different E:T ratios. Error bars represent the means ± SDs from three independent experiments. (F) and (G) Activation (CD25^+^CD69^+^) and degranulation (CD107a^+^) were measured after stimulation for 24 h and 6 h, respectively. (H) Western blot quantification of CD3ζ and CD3ζ pTyr142 in lysates from CAR-T cells after stimulation for 1 h. The fold change in expression of CD3ζ pTyr142 compared with that of CD3ζ with band intensity is reported as the means ± SDs from three independent experiments. (H) CAR-T cells were stimulated with fresh target cells every 5 days. Killing of the added target cells before every restimulation timepoint was determined via flow cytometry. On day 10 after stimulation, LAG-3 and PD-1 expression on CAR-T cells was measured (I). The data are presented as the means ± SDs from three independent experiments. All the statistical significance was calculated using the Newman-Keuls multiple comparison test: *P < 0.05, **P < 0.01, ***P < 0.001, and ns: not statistically significant (≥ 0.05).

Next, we explored the efficacy of LoopCAR-1 compared to that of monospecific CAR-T cells. As expected, LoopCAR-1 exhibited overall improvement against all tumor cells, whereas the monospecific CAR-T cells induced specific cytotoxicity against only the corresponding antigen-bearing leukemia cells (Fig. S7A). Notably, compared with the combination of monospecific CAR-T cells, LoopCAR-1 demonstrated greater efficiency, confirming that the architecture of LoopCAR-1 preserved the optimal steric activity of both binding domains. The cytokine release results further supported these findings (Fig. S7B). LoopCAR-1 also exhibited enhanced antitumor activity in primary B-ALL cells (Fig. S7C and S7D), indicating that the optimal configuration of the antibodies in the loop structure of the bispecific CAR allowed for efficient antigen-dependent signaling and activity.

To test *in vivo* antitumor efficacy, we examined CAR-T cells in xenograft models established with Nalm6 cells (CD19^+^CD22^+^) (Fig. S8A). Notably, LoopCAR-1 did not exhibit compromised antileukemic efficacy *in vivo*, and its kinetics were similar to those previously reported for CD19 CAR and CD19/CD22 CAR (Fig. S8B and S8C). Mice treated with LoopCAR-1 did not exhibit body weight loss and thus presented good safety profiles (Fig. S8D). As expected, the survival of mice treated with LoopCAR-1 was significantly prolonged (Fig. S8E). Additionally, the serum cytokine levels were significantly greater in the mice treated with LoopCAR-1 than in those treated with the CD22 CAR or the CAR combination (Fig. S8F). Collectively, these results suggested that LoopCAR-1 and LoopCAR-4 could achieve *in vivo* CD19^+^CD22^+^ tumor clearance activity comparable to that of the CD19 CAR.

Clinical evidence highlights the failure of CAR-T-cell therapies in leukemia patients with the downregulation or loss of B-cell antigens (Schneider et al., 2021). To explore the *in vivo* capacity of these strains to overcome antigen escape in a clinically relevant model of CD19 or CD22 resistance, we further evaluated LoopCAR-1 in an immune evasion model established with CD19- or CD22-knockout Nalm6 cell variants (1:1 ratio) (Fig. 2A). Consistent with the previous results, LoopCAR-1 significantly suppressed the growth of heterogenous tumors, promoted sustained disease remission (Fig. 2B and 2C), prolonged survival (Fig. 2E), increased serum cytokine levels (Fig. 2F), and did not cause weight loss (Fig. 2D). In contrast, the CD19 CAR or CD22 CAR alone failed to control the tumor burden. Notably, compared with LoopCAR-1, LoopCAR-4 and the combined monospecific CAR-T cells were ineffective at decreasing the tumor burden, underscoring the importance of structural optimization of LoopCAR-1, especially with respect to antibody and linker selection. We further tested the activity of LoopCAR-1 in another model (1:1:1 ratio) that mimics relapse in clinical practice. We obtained data consistent with previous data (Fig. S9A–F). Taken together, these results demonstrated that the LoopCAR-1 construct elicits an optimal CAR-T-cell response against leukemia, with decreased expression of single or dual antigens, which could prevent immune escape and tumor relapse associated with monospecific therapy. The enhanced efficacy of LoopCAR-1 over the combination of CD19 CAR and CD22 CAR highlights the advantages of this strategy in generating structurally optimized bispecific CAR-T cells.

**Figure 2.**
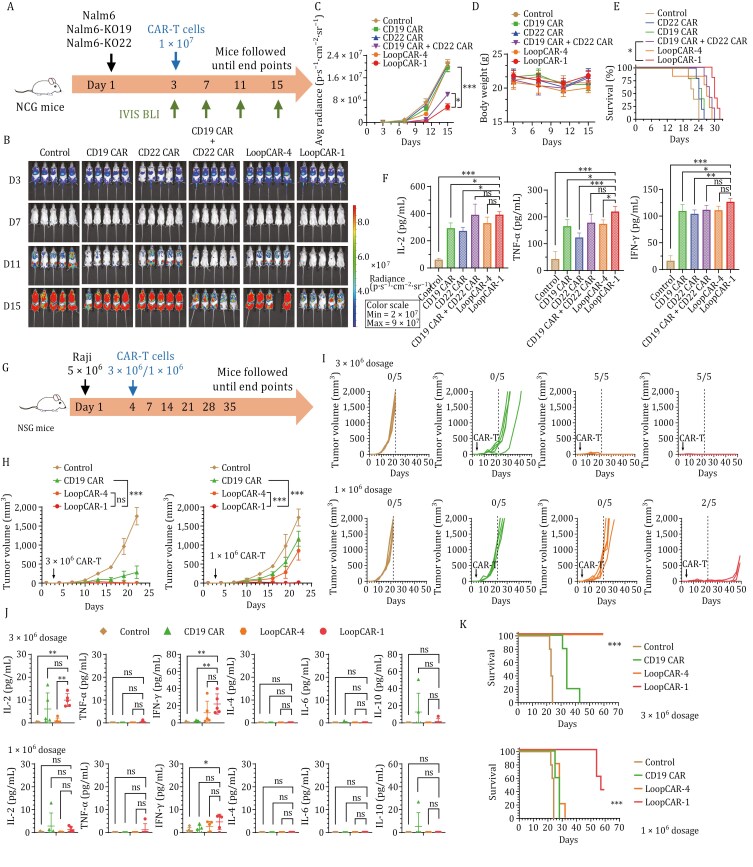
LoopCAR-1 demonstrated superior dual antigen targeting *in vivo* efficacy in a model of heterogenous leukemia and refractory lymphoma. (A) Scheme showing the experimental design. A mixture of luciferase-expressing Nalm6, Nalm6-KO19, and Nalm6-KO22 cells at a 1:1:1 ratio was used to establish the immune escape model. Three days after tumor implantation, the mice (*n* = 5/group) were i.v. administered 1 × 10^7^ corresponding CAR-T cells. The results were similar in two independent experiments. (B) Representative bioluminescence images of mice treated with different CAR-T cells. The colors indicate the intensity of luminescence (red, highest; blue, lowest). (C) Average radiance quantification (p/s/cm^2^/sr) of the luminescence is shown. Statistical significance was calculated using Dunnett’s multiple comparisons test. (D) Body weight was monitored during treatment. (E) Survival curves of mice treated with the indicated CAR-T cells. The log-rank (Mantel-Cox) test was used to calculate significance. (F) Serum cytokine levels were evaluated via ELISA 24 h after CAR-T-cell infusion. The data are presented as the means ± SDs. *P < 0.05, **P < 0.01, ***P < 0.001, and ns: not statistically significant (≥0.05). (G) Scheme showing the experimental design. The NSG mice were s.c. inoculated with 5 × 10^6^ Raji cells per mouse on study day 0. Three days after tumor implantation, the mice (*n* = 5/group) were i.v. administered 1 × 10^6^ or 3 × 10^6^ CAR-T cells. The results were similar in two independent experiments. (H and I) Average tumor growth in the two dosage groups. Tumor volume was measured and calculated using the formula: Volume = (length × width^2^)/2. Data are presented as the mean ± *SD*. Statistical significance for average tumor growth was calculated with two-way ANOVA and Tukey’s multiple comparisons. (D) Individual tumor volumes are plotted. The ratios indicate the number of tumor-free mice. (J) Th1 (IL-2, IFN-γ, TNF-α) and Th2 (IL-4, IL-6, IL-10) cytokine release into the serum was evaluated 24 h after CAR-T-cell infusion using a BD™ Cytometric Bead Array (CBA) Human Th1/Th2 Cytokine Kit. Asterisks indicate statistical significance using the Newman-Keuls multiple comparison test. (K) Survival curves of mice treated with the indicated CAR-T cells. The log-rank (Mantel-Cox) test was used to calculate significance. *P < 0.05, **P < 0.01, ***P < 0.001, and ns: not statistically significant (≥0.05).

Next, we evaluated the efficacy of CAR-T-cell therapy against clinically relevant PDX models of B-ALL (PDX Pt #7) (Fig. S10A). All the CAR-T cells demonstrated the ability to control the disease in the early stages after infusion (Fig. S10B). However, as the disease progressed, mice treated with either loop CARs or a mixture of monospecific CARs did not exhibit complete eradication of primary B-ALL cells. Significantly, the LoopCAR-1-treated group exhibited enhanced antileukemic activity during the later stages, as evidenced by the prolonged persistence of T cells in the peripheral blood compared to that of other CAR-T-cell therapies (Fig. S10C). No body weight loss was observed (Fig. S10D). Treatment with LoopCAR-1 resulted in a greater overall percentage of survival and greater production of Th1 cytokines (Fig. S10E and S10F). Moreover, after relapse, leukemia cells maintained CD19 and CD22 expression, precluding CAR stress-mediated antigen loss (Fig. S10G). These results demonstrated that LoopCAR-1 resulted in stable therapeutic effects on B-ALL PDX models, suggesting that it is a promising candidate for clinical therapy.

Furthermore, we extended the indications to lymphoma. The initial objective response rates of lymphoma patients to CD19 CAR-T cells exceeded 80%, but more than half of the patients relapsed within 12 months (Schuster et al., 2019). To determine whether the observed activities of LoopCAR-1 in B-ALL translate to lymphoma models, we conducted *in vitro* comparisons and found that, compared with those of CD19 CAR-T cells and LoopCAR-4, LoopCAR-1 exhibited markedly enhanced lymphoma killing and cytokine release activity (Fig. S11A and S11B). We further assessed the *in vivo* efficacy of these CAR-T-cell candidates in Raji lymphoma xenograft models (Fig. 2G). Consistent with the *in vitro* data, LoopCAR-1 showed significantly greater sensitivity to lymphoma than did LoopCAR-4, highlighting the substantial clinical potential of LoopCAR-1 (Fig. 2H and 2I). A significantly greater level of Th1 cytokines, prolonged survival, and stable body weight indicated that LoopCAR-1 not only provided enhanced efficacy but also ensured safety (Figs. 2J, 2K, S12A and S12B). Taken together, these data suggest that, in combination with synergistic antigen targeting activity, LoopCAR-1 has the potential to induce complete elimination of refractory lymphoma, leading to significantly improved *in vivo* efficacy compared with that of the CD19 CAR-T-cell and clinically active CD19/CD22 CAR-T-cell strategies.

Relapse has emerged as a significant challenge, impacting the long-term durability and efficacy of B-ALL and lymphoma treatment (Song et al., 2019). At present, CD19/CD22 CARs with rapid clinical progress include AUTO3 and Boom-configured CD19/CD22 CAR, which belong to the typical dual-receptor co-transducing CAR and tandem CAR structures, respectively. Despite the high initial response rate in their clinical trial, a substantial proportion of patients eventually had tumor recurrence. Currently, there are no comprehensive studies on the design of CD19/CD22 CARs exploring the adjustment of the spatial configuration between CAR-T cells and target cells. We believe that further efforts are needed to optimize the structure of CD19/CD22 CAR. Notably, this study addressed this gap by systematically highlighting the importance of the structural design and optimization of bispecific CARs for enhancing the ability to suppress heterogenous tumor growth through side-by-side comparisons with clinically active CAR-T cells or coadministration of monospecific CAR-T cells. The steric availability, structural aspects, and functional aspects of the target epitope need to be considered in CAR design. Previous studies by Qin et al. highlighted the significance of membrane-binding locations and linker types in influencing CAR functionality (Qin et al., 2018; Zanetti et al., 2022). Similarly, we found that the β-stranded linker was more efficient at displaying a nanobody with a compact domain than was scFv. In addition, we further extended the same Loop structure to solid tumor therapy to construct Her2/IGF1R LoopCAR, and obtained better anti-tumor activity than the classical tandem loop structure (data not shown), suggesting that this structure may be widely applicable.

In conclusion, we developed the novel bispecific CD19/CD22 LoopCAR-1, which has distinct advantages in enhancing CD22-redirected signaling compared with that of other loop CD19/CD22 CARs. Our findings suggest that LoopCAR-1 could be a promising therapeutic option for B-ALL or lymphoma patients with CD19 or CD22 expression. Further clinical translation and testing of this novel strategy are warranted to evaluate its potential efficacy.

## Supplementary data

Supplementary data is available at https://doi.org/10.1093/procel/pwae034.

pwae034_suppl_Supplementary_Figures_S1-S12_Tables_S1-S2

## Notes

Authors declare no competing interests.

The work was supported by the National Key R&D Program of China (2019YFA0906100 and 2019YFA0904200 to Y.J.C.), the Shenzhen Science and Technology Innovation Program (JCYJ20180504165501371 to Y.J.C.), the National Natural Science Foundation of China (32171464 and 81872783 to Y.J.C.), the Prevention and Control of COVID-2019 Research Program in University of Guangdong Province (2020KZDZX1176 to S.Z.), the Shenzhen Basic Research Program (JCYJ20180507182203049 to S.Z.) and the Shenzhen Key Laboratory Foundation (ZDSYS20200811143757022 to S.Z.). Chinese patent “Construction of A Novel Bispecific Chimeric Antigen Receptor and Uses Thereof,” with the application number CN202210126836.0, is related to the present work.

L.Z., S.L., and Y.J.C. designed the studies, analyzed the data, and wrote the manuscript; L.Z. and S.L. performed the experiments; X.W. and L.S. assisted in the animal experiments; X.Q. assisted in the cytokine detection; Q.G. assisted in the visual abstract; J.-s.Z. supported alpaca immunization and the screening of CD22-targeted nanobodies; J.L. provided protocols and advice on lentivirus production and T-cell transduction; Z.-l.L. and Z.G. provided the B-ALL patient samples; H.Z. and J.F. provided advice on establishing the B-ALL and lymphoma models; Y.S. was responsible for the GMP production and preparation of lentivirus and CAR-T cells; S.Z. assisted in the design of the animal study; and all the authors reviewed the manuscript, approved the final version and agreed to submit the manuscript for publication.

Additional informed consent was obtained from all patients for whom identifying information is included in this article. All institutional and national guidelines for the care and use of laboratory animals were followed.

All the authors reviewed the manuscript, approved the final version, and agreed to submit the manuscript for publication. For original data, please contact joshuacao@pku.edu.cn.
