# Supplementary material for: A novel loop-structure-based bispecific CAR that targets CD19 and CD22 with enhanced therapeutic efficacy against B-cell malignancies
Source: Protein Cell. 2024 Jun 1;16(3):227–31. doi: 10.1093/procel/pwae034 (PMC11891134; doi:10.1093/procel/pwae034)
Supplement: pwae034_suppl_Supplementary_Figures_S1-S12_Tables_S1-S2 [file pwae034_suppl_supplementary_figures_s1-s12_tables_s1-s2.pdf]

Materials and Methods

Experimental Design

The aim of this study was to identify the optimal structure of a CD19/CD22 bispecific CAR-T-cell for treating antigen-heterogeneous cancers and to address the limitations of bispecific CAR-T cells with inefficient CD22-redirected activity in clinical settings. We hypothesized that an optimal combination of antibodies and linkers would enhance immune synapse formation, thereby enabling optimal dual-targeted antitumor activity and improving the efficacy of overcoming immune escape. To investigate these abilities, we generated an immune escape model using Nalm6 and CRISPR-edited Nalm6-knockout cell lines. The efficacy of CARs against the B-ALL PDX model was evaluated using primary B-ALL patient samples. Sample sizes were determined based on previous experiments and a literature review. The number of biological (n) and technical replicates is indicated in the figure legends. Before the administration of CAR-T cells, the animals were randomized based on the mean luminescence signal of luciferase. *In vivo* studies excluded patients who died unrelated to tumors. The data analysis was performed in an unblinded manner.

Clinical data of B-ALL patients

Peripheral blood or bone marrow samples were collected from patients with B-ALL after providing informed consent. Samples from B-ALL patients used in the studies reported here were selected based on the availability of viably frozen PBMCs. Clinical and laboratory data were collected from electronic medical records. Table S2 shows the clinical and biological features of the B-ALL samples used.

Cell lines and culture conditions

The cell line K562 and Nalm6 were purchased from ATCC. These suspension cell lines were cultured in RPMI1640 complete media (Hyclone, SH30255.01) supplemented with 10% heat-inactivated fetal bovine serum (FBS) (Genstar, C511-10), 1% penicillin, 1% streptomycin, 0.1mM non-essential amino acids, 6mM L-glutamine and 1mM sodium pyruvate. Human T-cells were cultured and expanded in AIM-V media including 300IU/mL IL-2 (Genscript, Z00368) and 5% FBS. Lentivirus producing cell line 293FT was purchased from Thermo Fisher Scientific and maintained in DMEM high glucose complete media.

Construction of CAR expression vectors and T-cell transduction

For CAR construction, we utilized previously validated targeting moieties, including an anti-CD19 scFv (FMC63) and a CD22-specific scFv (M971) or nanobody (Nb25) (Haso, Lee, Shah, et al., 2013,Zhao, Li, Wei, et al., 2022). To create a comprehensive set of CARs, we designed CD19/CD22 CARs with different bispecific binding domains as follows: TanCAR-1 or TanCAR-2 (in which Nb25 and FMC63 were connected in sequential order by a flexible and protease-resistant G_4S linker or a rigid long hinge lama linker, respectively); LoopCAR-1 (in which Nb25 was incorporated between VL and VH of FMC63 by a β-stranded linker); LoopCAR-2 (in which Nb25 was incorporated between VL and VH of FMC63 by a G_4S linker); LoopCAR-3 (in which M971 was incorporated between VL and VH of FMC63 by a β-stranded linker); and clinically active LoopCAR-4 (in which M971 was incorporated between VL and VH of FMC63 scFv by a G_4S linker) (Qin, Ramakrishna, Nguyen, et al., 2018,Spiegel, Patel, Muffly, et al., 2021). The linker sequences are listed in Table S1. All the monospecific or bispecific antigen-binding regions were incorporated into a second-generation CAR construct harboring the human CD8 hinge, CD8 transmembrane, 4-1BB costimulatory and CD3ζ activation domains. This design closely resembles the second-generation CARs used by June and coworkers for CART-19 cells. CAR-expressing viral particles were generated by transfecting 293FT cells with lentivirus packaging plasmids (pMDLg/pRRE, pRSV-rev and pVSV-G) and the pELPS plasmid using Lipofectamine 2000 (Thermo Fisher Scientific, 11668019) according to the manufacturer's instructions (Frey, Shaw, Hexner, et al., 2020). Virus-containing supernatants were collected 48 hours after transfection.

Human peripheral blood mononuclear cells (PBMCs) were isolated from healthy donors using a standardized density gradient technique (Ficoll-Paque, GE Healthcare, 17-1440-03) and incubated in tissue culture flasks for 1 hour to remove adherent cells. PBMCs were then activated with CD3/CD28 Dynabeads (Thermo Fisher Scientific, 11141D) for 48 hours in X-VIVO^{15} (with 5% FBS) media supplemented with 300 IU/mL recombinant IL-2. Activated T cells were transduced with the CAR-containing lentivirus at a multiplicity of infection of 3 in the presence of 6 μg/mL polybrene and 300 IU/mL IL-2 in novonectin-treated 24-well plates. The plates were centrifuged at 1000 × g for 30 minutes at 25℃ and then incubated at 37℃. The CD3/CD28

Dynabeads were removed 6 days after transduction, and the CAR expression level was subsequently determined via flow cytometry.

Flow cytometry

The expression of CAR-transduced T-cells was detected using PE conjugated recombinant CD19 protein and APC conjugated recombinant CD22 protein. Fluorescently labeled antibodies: anti-human CD3-Pacific Blue (HIT3a), anti-human CD45-FITC (HI30), anti-human CD10-APC-Cy7 (HI10a), anti-human CD19-APC (HIB19), anti-human CD22-PE (HIB22) and anti-human CD19-Pacific Blue (SJ25C1) were obtained from Biolegend. Tumor cells were stained with anti-human CD19-APC and anti-human CD22-PE in fluorescence-activated cell sorting (FACS) stain buffer to identify the expression level of CD19 and CD22 on cell surface. Primary patient samples were stained with anti-human CD10-APC-Cy7, anti-human CD19-Pacific Blue and anti-human CD22-PE. For PDX model, samples were stained with anti-human CD45-FITC, anti-human CD3-Pacific Blue, anti-human CD10-APC-Cy7, anti-human CD19-APC and anti-human CD22-PE. Dead cells in all studies were excluded by 7-AAD staining (BD BioSciences, 559925) before quantitative analysis. Flow cytometric analysis was performed on a flow cytometer (Attune NxT, Thermo Fisher Scientific) and all the flow cytometry data was analyzed with FlowJo software.

Generation of CD19- or CD22-knockout Nalm6 cell lines

CD19- or CD22-knockout (KO) Nalm6 cells (Nalm6-KO19 and Nalm6-KO22) were obtained by CRISPR/Cas9-mediated gene editing of the parental Nalm6 cell lines. Lenti-CRISPR plasmids were designed to deliver Cas9 and specific guide RNA (gRNA) for CD19 antigen (AAGCGGGGACTCCCGAGACC on exon 3), or gRNA for CD22 antigen (GGATCATCAGAAGACCCCCC on exon 6). 1 μg of Lenti-CRISPR plasmid was electroporated into 0.2×10^6 Nalm6 cells using Neon™ transfection system (Thermo Fisher Scientific). All KO cells were detected for CD19 and CD22 surface expression by flow cytometry and western blot to confirm genotypic alterations as described in a previous publication.(Zhao, Li, Wei, et al., 2022)

Generation of K562 and Nalm6 variants with different antigen densities

We generated a panel of stable genetically engineered cells, including different CD19- or CD22-expressing K562 cells (K562-CD19 and K562-CD22). The genes encoding full-length of CD19- or CD22-antigen were synthesized by Genscript and cloned into the pLV lentiviral plasmid. CD19-expressing or CD22-expressing viral particles were generated by the transfection of HEK293FT cells with lentivirus packaging plasmids using lipofectamine 2000 (Thermo Fisher Scientific, 11668019) according to manufacturer's instructions. Virus-containing supernatants were collected 48 hours after transfection. K562 cells were then transduced with the lentivirus supernatant and sorted by flow cytometry for the variants with different CD19- or CD22-expression levels as described in a previous publication.(Zhao, Li, Wei, et al., 2022)

***In vitro* cytotoxicity, activation and cytokine release**

CFSE-labeled tumor cells ($CFSE^+$) were cocultured with CAR-T cells for 24 hours at different E:T ratios. After incubation, the dead cells were excluded by 7-AAD staining. CAR-T-cell-mediated cytotoxicity was evaluated using a flow cytometer by counting the residual live target cells (identified as 7-AAD^-CFSE^+). For the primary patient samples, the cells were stained with anti-human CD3-Pacific Blue, anti-human CD10-APC-Cy7 and 7-AAD after incubation, and live patient tumor cells were identified as $7\text{-AAD}^-hCD3^-hCD10^+$ cells, as described in a previous publication (Zhao, Li, Wei, et al., 2022). The activation and cytokine secretion levels after 24 hours of coculture at an E:T ratio of 1:3 were measured via flow cytometry and human IL-2, IFN-γ and TNF-α (Thermo Fisher Scientific) test kits.

***In vitro* proliferation**

For proliferation assay, CAR-T cells were labeled with CFSE (Biolegend, 423801) and then incubated with target cells at an E: T ratio of 1: 1. Six days later, the cell number was analyzed by flow cytometry. All the tests were conducted in duplicate and results are shown as means ± SDs.

Binding assay

Binding affinity of CD22 nanobody (Nb25) to CD22 antigen were measured by ELISA: The full length CD22-Fc or truncated CD22-Fc were expressed in FreeStyle HEK293 suspension cells by transient transfection and purified using Protein G chromatography. And Then, 10ng/well of

CD22 antigen were coated on 96-well ELISA plate at 4℃, followed by blocking with 5% milk in Tris-NaCl (pH 8.0) for 2 hours at room temperature. After washing with 0.05% Tween-20 in PBS, serial dilutions of CD22 nanobody (Nb25) were added and incubated for 2 hours at room temperature, and followed with HRP-labeled anti-Myc tag antibody (Biolegend, 626803) for 2 hours at room temperature. After washing, 3,3,5,5-Tetramethylbenzidine (TMB) substrate were incubated and reaction were stopped by 0.2M H_2SO_4. Fluorescence was quantified using Cytation 5 Cell Imaging Multi-Mode Reader (Agilent Technologies) with excitation at 450nm. The data are plotted and analyzed in GraphPad Prism software by nonlinear regression in the model of logarithm (agonist) vs. response.

Binding affinity of the two loop CAR-T cells to CD22 antigen were measured by flow cytometry to analysis the relative fluorescence intensity. CAR-T cells were incubated with full length CD22-Fc fusions or truncated CD22 (d6-d7)-Fc fusions at different concentrations for 1 hour on ice. After washes, the cells were then stained with Alexa Fluor 647 (AF647) conjugated anti-human IgG antibody (Invitrogen, A21445) for 30 minutes on ice prior to data acquisition on flow cytometer.

Immunofluorescence analysis of immune synapse formation

Immunofluorescence-based conjugation studies were performed based on Nalm6-KO19 and Nalm6-KO22 cells (stably transduced with GFP) and the four loop CAR-T cells. 1×10^5 CAR^+ cells were incubated with 5×10^4 Nalm6-KO19 or Nalm6-KO22 cells in RPMI1640 complete media, and then incubated onto poly-L-lysine-coated coverslips at incubator for 1 hour. After centrifuge at 1500rpm for 5 minutes, cells were gently washed with PBS and fixed with 4% paraformaldehyde. To detect intracellular proteins, cells were permeabilized with 0.2% Triton X-100 for 10 minutes. For immunofluorescence, cells were first blocked with BSA blocking reagent (5% BSA in PBS) and then stained with primary rabbit anti-human PKC θ Biotin conjugated antibody (Signalway Antibody, C33151). After gently washing, cells were stained with PE-Streptavidin (BioLegend, 405204). To identify nucleus, cells were stained with Hochest (Solarbio, c0031). And fluoroshield quencher (Sigma-Aldrich, F6182) were used in the final step to avoid fluorescence quenching. Finally, conjugations were measured by a laser scanning confocal microscope (A1R, Nikon) under oil immersion objective (100×) at room temperature.

Conjugates were analyzed by ImageJ software and identified using a gating strategy to include the red areas between tumor and CAR-T cells.

Degranulation

CD107a staining were performed after incubation of CAR-T cells and tumor cells for 6 hours in 24-well plates at a ratio of 1:1. CD107a-APC antibody (1μg/mL) (BioLegend, 328612) were added at the time of stimulation and monensin (Sigma, 2μM) were added after 1 hour of stimulation.

Phosphorylation and western blotting

CAR-T cells were resuspended at a concentration of 2×10^{7} cells/mL. Cells were then incubated with target cells at an E: T ratio of 1:3 for 1 hour at incubator. And then the cells were quickly washed twice using ice-cold PBS and lysed in a strong lysis solution (Mei5bio, MF072-01) supplemented with protease inhibitors (Mei5bio, MF182-plus-01). Lysates were centrifuged at 10,000g and 4 ℃ for 10 minutes.

Lysate was quantified using BCA kit (Elabscience, E-BC-K318-M) and equal masses of protein lysate were loaded into 12% SDS-PAGE gels. After protein transfer onto nitrocellulose membranes, membranes were blocked with 5% nonfat dry milk. Membranes were stained with primary and secondary antibodies diluted in blocking buffer. The following antibodies were used: anti-human CD3ζ (Santa Cruz Biotechnology, sc-1239), anti-human CD3ζ $pTyr^{142}$abcam, AB68235), anti–mouse IgG-HRP (TransGen Biotech, HS201-01) and anti–rabbit IgG-HRP (TransGen Biotech, D110058-0025). Antibody dilutions ranged from 1:10,000 to 1:2000. Band intensities were quantified using ImageJ and the intensities of CD3ζ $pTyr^{142}$ was normalized to the intensities of CD3ζ.

Multiple stimulation assay

T cells were cocultured with GFP^{+} targeted cells at an initial E: T ratios of 3: 1. After 5 days of incubation, the dead cells were excluded by 7-AAD staining. CAR-T cell-mediated cytotoxicity before every re-stimulation timepoint was evaluated before using a flow cytometer by counting the residual live target cells (identified as $7\text{-}AAD^{-}GFP^{+}$). Every five days, T cells were

re-stimulated with the same amount of tumor cells as initial added. The expression levels of PD-1 (BioLegend, 329906) and LAG-3 (BioLegend, 369308) on day 10 were measured by flow cytometry staining as described above. For the phenotype, we stained for CD45RO (BioLegend, 304242) and CCR7 (BioLegend, 353214) markers by flow cytometry and the percentage of $CCR7^{-}CD45RO^{-}$ (effector) cells was statistically analyzed.

***In vivo* efficacy study**

All *in vivo* experimental procedures were approved by the Peking University Shenzhen Graduate School Animal Care and Use Committee and performed according to the national and international guidelines for the humane treatment of animals. Female NOD-Prkdc^{em26Cd52}Il2rg^{em26Cd22}/NjuCrl (NCG) mice were purchased from Gem Pharmatech. Female NOD-Prkdc scidIl2rg em1/Smoc (NSG) mice were purchased from Shanghai Model Organisms.

In the Nalm6 xenograft lymphoma model, NCG mice were i.v. injected with Nalm6 cells stably transduced with firefly luciferase at 1×10^6 cells per mouse on study day 0. For the heterogeneous antigen model, a mixture of tumor cells comprising luciferase-expressing Nalm6-KO19 and Nalm6-KO22 cells at an equal proportion was used to maintain immune escape from two phenotypes, and luciferase-expressing Nalm6, Nalm6-KO19 and Nalm6-KO22 cells at a 1:1:1 ratio were used to maintain immune escape from the three phenotypes. Tumor burden was monitored by bioluminescence imaging using an IVIS Spectrum (PerkinElmer). Three days after tumor implantation, the mice were i.v. administered 10×10^6 CAR-T cells. Twenty-four hours after CAR-T-cell administration, proinflammatory cytokines in the serum were detected as described above.

For the Raji lymphoma model, NSG mice were s.c. inoculated with 5×10^6 Raji cells per mouse on study day 0. Three days after tumor implantation, the mice were intravenously (i.v.) administered either 1×10^6 or 3×10^6 CAR-T cells. Th1/Th2 cytokine levels in the serum were measured 24 hours after CAR-T-cell administration. Tumor volume was measured and calculated using the formula: Volume = (length $\times$ width^2)/2 until it reached a maximum of 20 mm in either length or width or until the total volume reached 2000 mm^3, at which point the animals were euthanized.

PDX model

NCG mice were i.v. engrafted with 5×10^6 tumor cells from a primary B-ALL patient sample (B-ALL Pt #7). After B-ALL cells were implanted in peripheral blood (for 32 days), the mice were distributed into groups based on the tumor burden, and then, 30×10^6 CAR-T cells were intravenously (i.v.) injected. The tumor burden and proportion of CAR-T cells in peripheral blood were monitored weekly by flow cytometry as described in a previous publication (Zhao, Li, Wei, et al., 2022). Twenty-four hours after CAR-T-cell administration, Th1/Th2 cytokine levels in the serum were measured.

Th1/Th2 cytokine measurement

The BD™ Cytometric Bead Array (CBA) Human Th1/Th2 Cytokine Kit II (BD Pharmingen, 551809) were used for cytokine detection according to the instructions. It was used to quantitatively measure IL-2, IL-4, IL-6, IL-10, TNF-α and IFN-γ cytokine levels in the serum. The complexes could be measured using flow cytometry to identify particles with fluorescence characteristics. The results are shown as the means ± SDs.

Statistical analysis

All the statistical analyses were conducted using GraphPad Prism version 8.4.2 software. The data are presented as the mean ± standard deviation (mean ± SD), and significance was determined using the Newman–Keuls multiple comparison test. Survival data are reported in Kaplan–Meier plots and were analyzed using the log-rank test.

References

Haso W, Lee DW, Shah NN, et al. Anti-CD22-chimeric antigen receptors targeting B-cell precursor acute lymphoblastic leukemia. *Blood*. 2013;**121**:1165-1174.

Zhao L, Li S, Wei X, et al. A novel CD19/CD22/CD3 trispecific antibody enhances therapeutic efficacy and overcomes immune escape against B-ALL. *Blood*. 2022;**140**:1790-1802.

Qin H, Ramakrishna S, Nguyen S, et al. Preclinical Development of Bivalent Chimeric Antigen Receptors Targeting Both CD19 and CD22. *Mol Ther Oncolytics*. 2018;**11**:127-137.

Spiegel JY, Patel S, Muffly L, et al. CAR T cells with dual targeting of CD19 and CD22 in adult patients with recurrent or refractory B cell malignancies: a phase 1 trial. *Nat Med*. 2021;**27**:1419-1431.

Frey NV, Shaw PA, Hexner EO, et al. Optimizing Chimeric Antigen Receptor T-Cell Therapy for Adults With Acute Lymphoblastic Leukemia. *J Clin Oncol*. 2020;**38**:415-422.

Table S1. Linker sequences used in CARs

Linkers	Amino acid sequences
G_4S linker	GGGGS
Long hinge lama linker	EPKIPQPQPKPQPQPQPQPQPKPQPKPEP
Beta-stranded linker (ascending peptide)	EETKKYQS
Beta-stranded linker (descending peptide)	SYTYNYEK
218 linker	GSTSGSGKPGSGEGSTKG

Table S2. Preparation and characterization of B-ALL patient samples

Patient	Sex	Age, y	Diagnosis	Disease status	% blasts in the blood
B-ALL Pt #1	M	14	Pre-B-ALL	De novo	94.85
B-ALL Pt #2	F	72	B-ALL	De novo	88.67
B-ALL Pt #3	M	37	B-ALL	De novo	86.9
B-ALL Pt #4	M	45	CML to Com-B-ALL	Relapse	35.11
B-ALL Pt #5	F	29	B-ALL	De novo	75.5
B-ALL Pt #6	F	52	B-ALL	De novo	32.0
B-ALL Pt #7	F	67	B-ALL	De novo	89.83

CML, chronic myelocytic leukemia; and Com-B-ALL, common B cell acute lymphoblastic leukemia; and Pre-B-ALL, precursor B cell acute lymphoblastic leukemia;

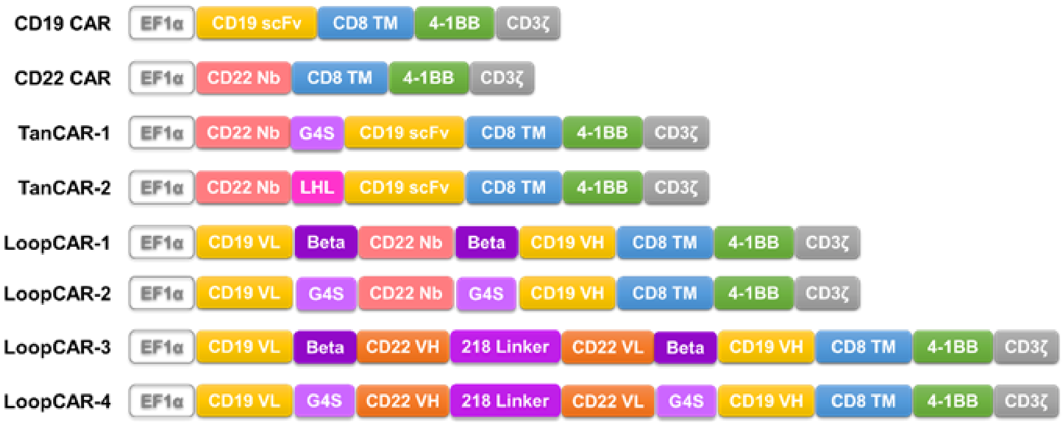

Figure S1. Schematic structures of CARs.

All the CARs containing the CD8 signaling sequence, corresponding binding domains, CD8 hinge region and transmembrane, 4-1BB costimulatory domain and CD3ζ signaling domain. For the bispecific binding domains, TanCAR-1 or TanCAR-2 represents Nb25 connected with FMC63 scFv by a flexible G_4S linker or a rigid long hinge lama linker; LoopCAR-1 is assembled by incorporating Nb25 between VL and VH of FMC63 scFv by β-stranded linker; LoopCAR-2 is assembled by incorporating Nb25 between VL and VH of FMC63 scFv by G_4S linker; LoopCAR-3 is composed of a targeting domain by incorporating M971 scFv between VL and VH of FMC63 scFv by β-stranded linker; and LoopCAR-4 is composed of a targeting domain by incorporating M971 scFv between VL and VH of FMC63 scFv by G_4S linker.

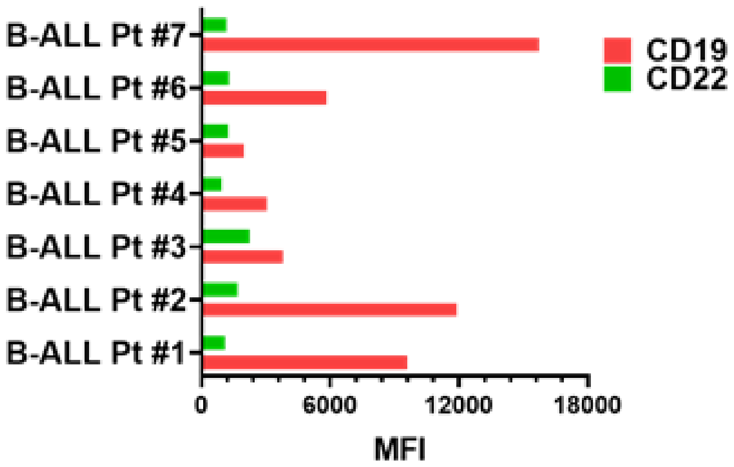

Figure S2. Characterization of B-ALL patient samples.

Quantitative analysis of CD19 and CD22 antigen expression levels in primary B-ALL patient samples by flow cytometry.

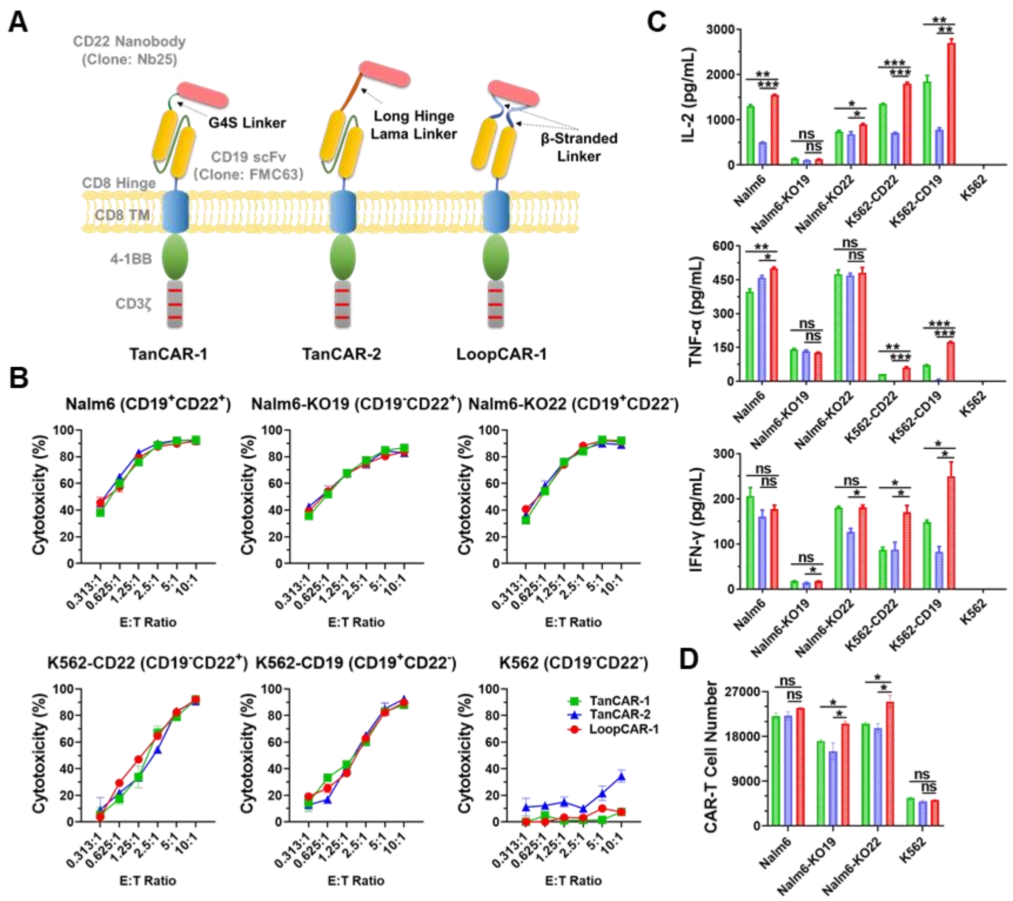

Figure S3. Development of bispecific CD19/CD22 CARs in tandem or loop structures

(A) Schematic structures of CD19/CD22 CARs. The targeting moiety of TanCAR-1 or TanCAR-2 represents Nb25 connected with FMC63 by a flexible G_4S linker or a rigid long hinge lama linker; the binding domain of LoopCAR-1 is assembled by incorporating Nb25 between VL and VH of FMC63 scFv by β-stranded linker; all the bispecific CARs contain CD19/CD22 binding domains, CD8 hinge region and transmembrane domain, 4-1BB costimulatory domain and CD3ζ signaling domain. (B) Cytotoxicity comparison of CD19/CD22 CARs against indicated tumor cells after 24-hours incubation at different E: T ratios. Error bars represent the means ± SDs from three independent experiments. (C) Inflammatory cytokines (IL-2, TNF-α and IFN-γ) released from CD19/CD22 CARs cocultured with the indicated target cells for 24-hours at an E: T ratio of 1: 3 in triplicate. The data are presented as the means ± SDs from three independent experiments. (D) Expansion of CD19/CD22 CARs after 6 days of incubation with indicated tumor cells at an E: T ratio of 1: 1. Error bars represent the means ± SDs from three technical replicates. All statistical significance was calculated using the Newman–Keuls multiple comparison test: *$P < 0.05$, **$P < 0.01$, ***$P < 0.001$, and ns: not statistically significant (⩾0.05).

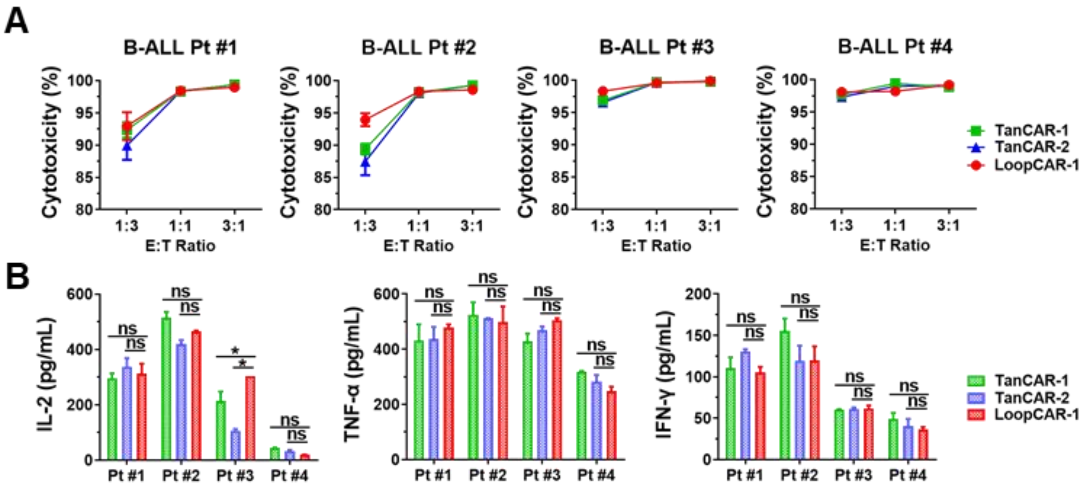

Figure S4. The comparison of bispecific CD19/CD22 CARs in tandem or loop structures.

(A) Cytotoxicity comparison of CD19/CD22 CARs against primary B-ALL patient tumor cells after 24-hours incubation at different E: T ratios. Error bars represent means ± SDs from three independent experiments. (B) Inflammatory cytokines (IL-2, TNF-α and IFN-γ) released from CD19/CD22 CARs cocultured with the primary B-ALL patient tumor cells for 24-hours at an E: T ratio of 1: 3 in triplicate. The data are presented as the means ± SDs from three independent experiments. And all statistical significance was calculated using the Newman−Keuls multiple comparison test: *$P < 0.05$, **$P < 0.01$, ***$P < 0.001$, and ns: not statistically significant (⩾0.05).

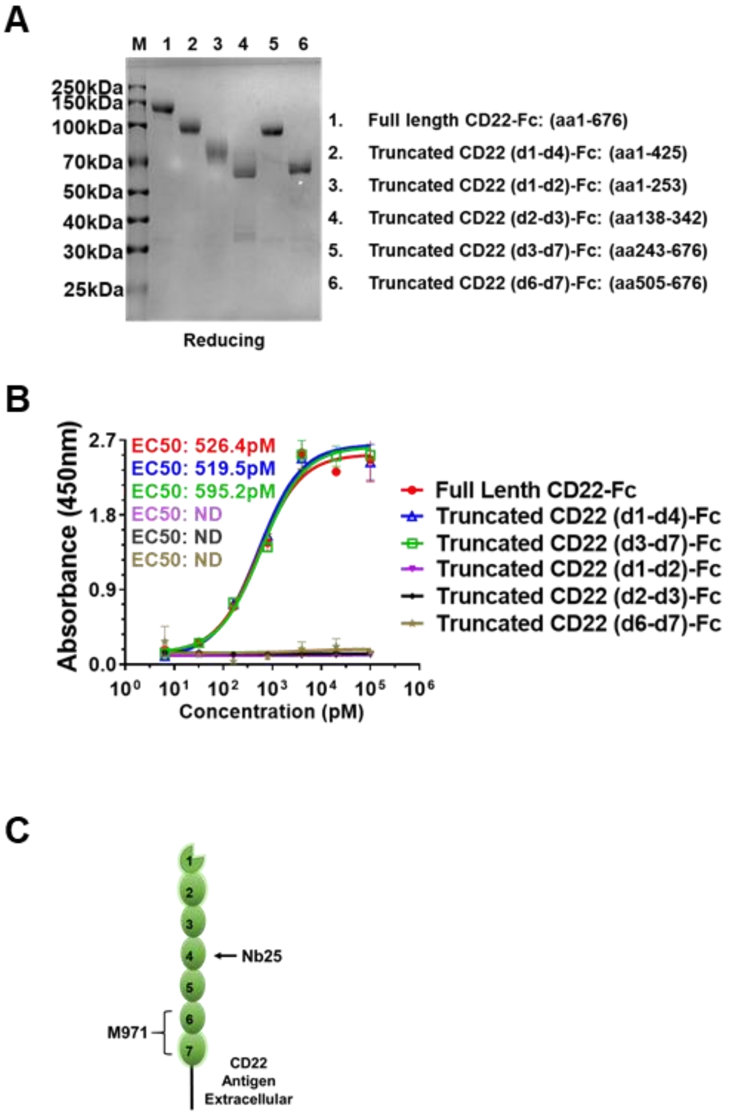

Figure S5. Validation of CD22-targeting antibody M971 and Nb25 antigen-binding epitopes. (A) SDS-PAGE analysis of full length or truncated CD22 antigen under reducing condition. (B) Binding activities of CD22 nanobody (Nb25) to CD22 antigen were detected by ELISA in triplicate. The data are presented as the means ± SDs from three independent experiments. ND: not determined. Error bars represent means ± SDs from three independent experiments. (C) Schematics of the binding sites of two CD22 antibodies, M971 scFv and Nb25 nanobody, that recognize the CD22 antigen. Nb25 nanobody recognizes distal epitopes on domain d4 of CD22, whereas M971 recognizes proximal epitopes on domain d6-d7 of CD22.

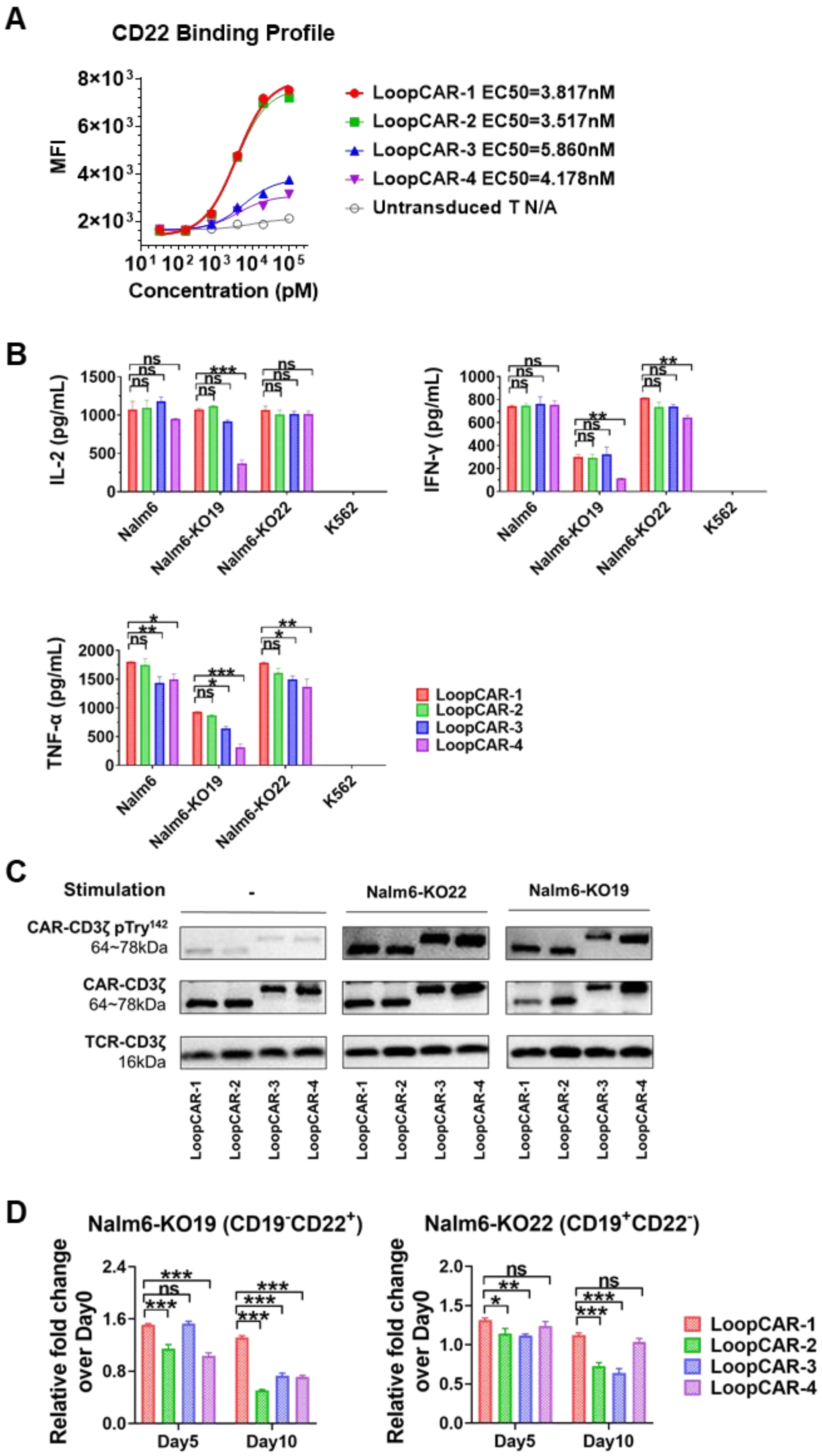

A
CD22 Binding Profile
MFI
Concentration (pM)
LoopCAR-1 EC50=3.817nM
LoopCAR-2 EC50=3.517nM
LoopCAR-3 EC50=5.860nM
LoopCAR-4 EC50=4.178nM
Untransduced T N/A
B
IL-2 (pg/mL)
IFN-γ (pg/mL)
TNF-α (pg/mL)
Nalm6
Nalm6-KO19
Nalm6-KO22
K562
LoopCAR-1
LoopCAR-2
LoopCAR-3
LoopCAR-4
C
Stimulation
Nalm6-KO22
Nalm6-KO19
CAR-CD3ζ pTry142
64~78kDa
CAR-CD3ζ
64~78kDa
TCR-CD3ζ
16kDa
D
Nalm6-KO19 (CD19-CD22+)
Nalm6-KO22 (CD19+CD22-)
Relative fold change over Day0
Day5
Day10

Figure S6. The *in vitro* efficay of LoopCAR-1 outperforms other Loop designs.

(A) The binding activities of CD22 antigens to different LoopCARs or T cells were detected by flow cytometry in triplicate followed with an APC-conjugated anti-FLAG tag second antibody. The mean ± SD of the MFI obtained from three independent experiments is indicated. (B) Inflammatory cytokines (IL-2, TNF-α and IFN-γ) released from the four loop-structure CARs cocultured with the indicated target cells for 24-hours at an E:T ratio of 1: 3 in triplicate. The data are presented as the means ± SDs from three independent experiments. And all statistical significance was calculated using the Newman–Keuls multiple comparison test: *P < 0.05, **P < 0.01, ***P < 0.001, and ns: not statistically significant (≥0.05). (C) CD3ζ and CD3ζ pTry142 of four LoopCARs in response to stimulation. LoopCAR-1, LoopCAR-2, LoopCAR-3, LoopCAR-4 CAR-T cells were stimulated for 1h at a 1:3 E:T ratio. CAR-CD3ζ pTry^{142}, CAR-CD3ζ, TCR-CD3ζ were measured by Western blotting. (D) Ratio of changes in the percentage of effector phenotypes of CAR-T cells at corresponding time points after stimulation. CAR-T cells were stimulated with fresh target cells every 5 days. The data are presented as the means ± SDs from three independent experiments. And all statistical significance was calculated using the Newman–Keuls multiple comparison test: *P < 0.05, **P < 0.01, ***P < 0.001, and ns: not statistically significant (≥0.05).

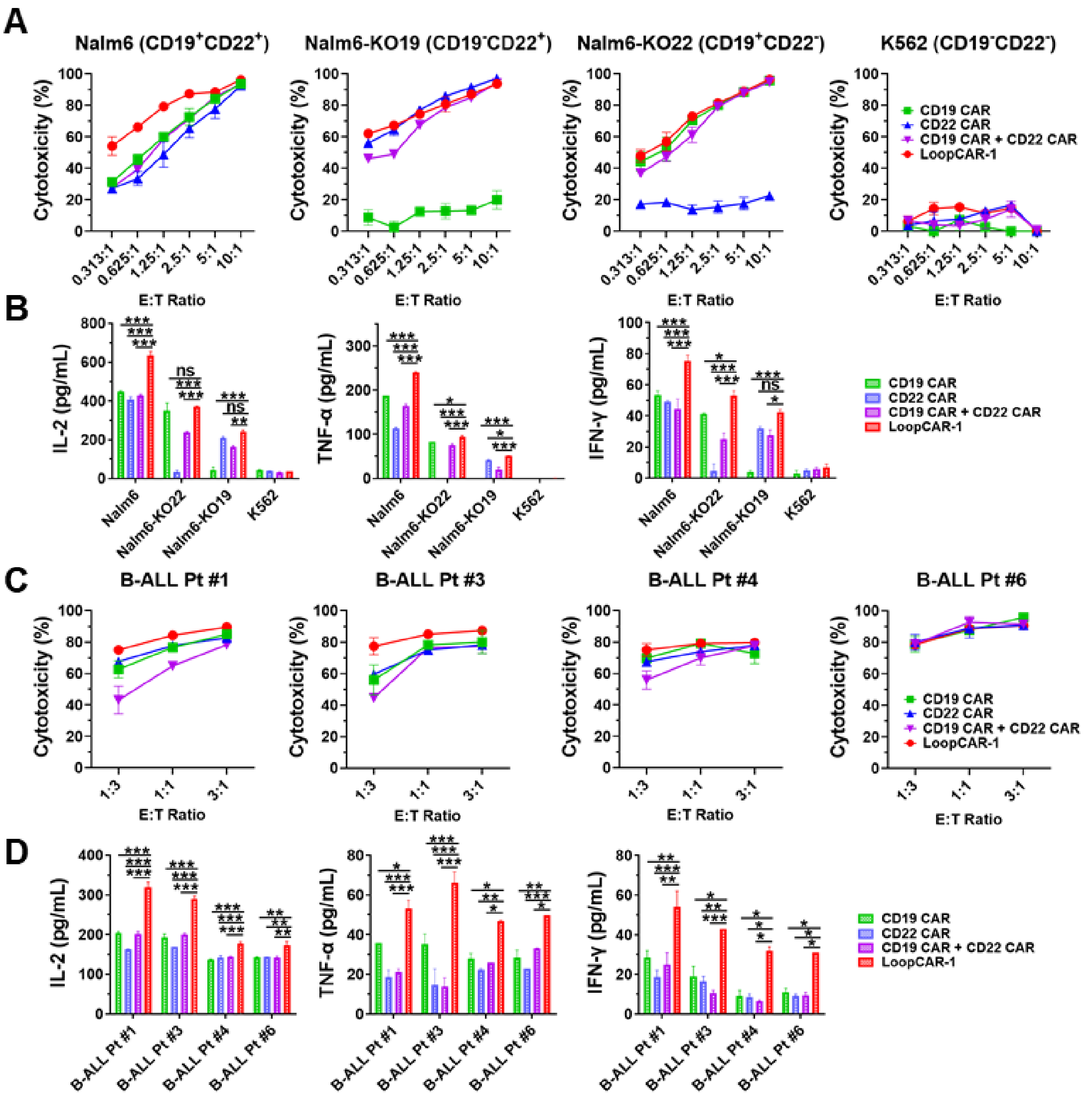

Figure S7. LoopCAR-1 exhibited superiority to monospecific CARs alone or in combination

Cytotoxicity comparison of LoopCAR-1, monospecific CARs alone or in combination against indicated tumor cell lines (A) or primary B-ALL patient samples (C) after 24-hours incubation at different E: T ratios. Error bars represent the means ± SDs from three independent experiments. Inflammatory cytokines (IL-2, TNF-α and IFN-γ) released from CAR-T cells cocultured with the indicated tumor cell lines (B) or primary B-ALL patient samples (D) for 24-hours at an E: T ratio of 1: 3 in triplicate. The data are presented as the means ± SDs from three independent experiments. Asterisks indicate statistical significance using the Newman−Keuls multiple comparison test. *P < 0.05, **P < 0.01, ***P < 0.001, and ns: not statistically significant (⩾0.05).

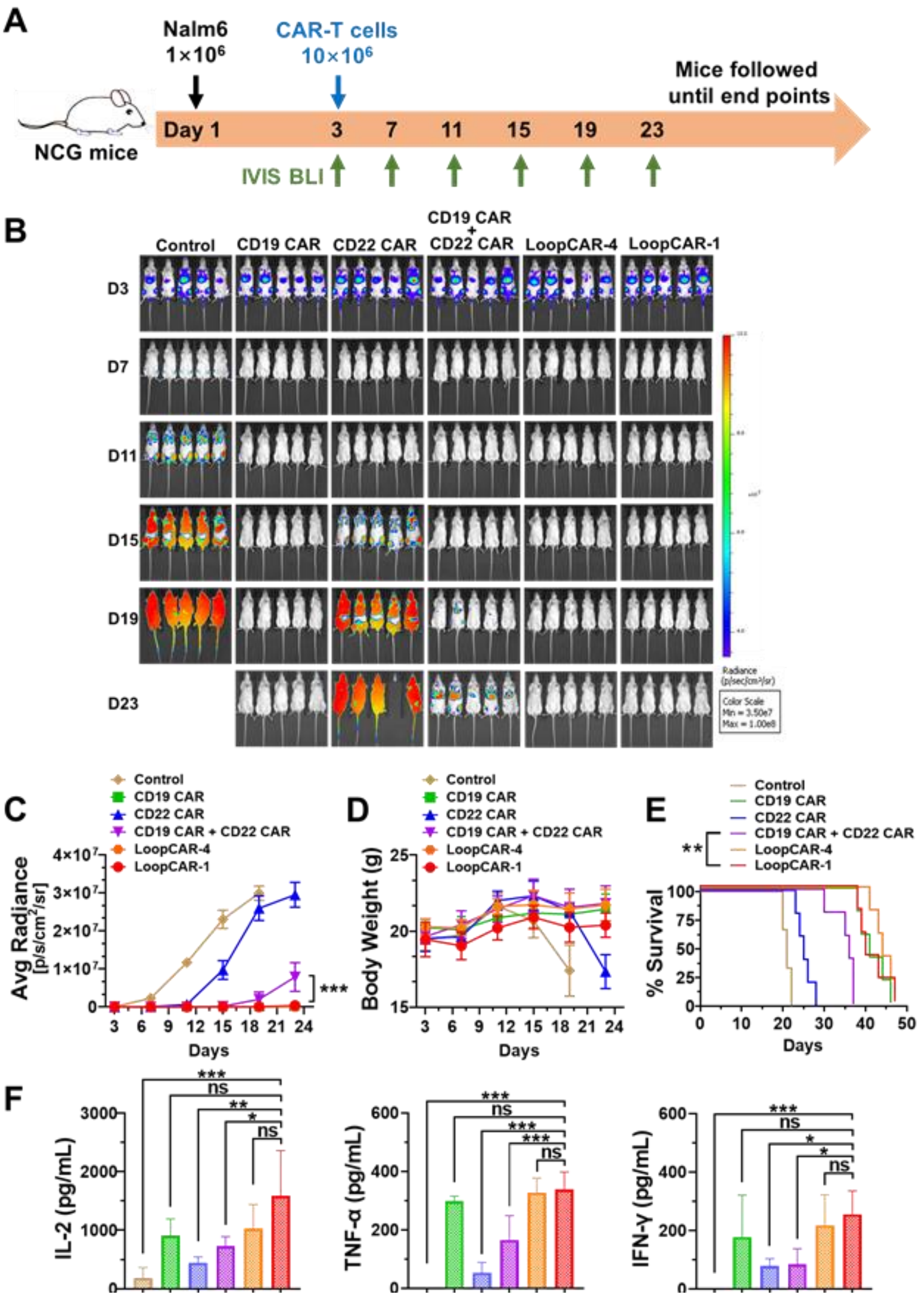

Figure S8. Loop structure of CARs retains the efficiency of CD19 CAR *in vivo*

(A) Scheme showing the experimental design. NCG mice (n = 5/group) were i.v. implanted with Nalm6 cells stably transduced with firefly luciferase at 1×10^6 cells per mouse on study day 0. Three days after tumor implantation, the mice were i.v. administered 10×10^6 corresponding CAR-T cells. Similar results were obtained in two independent experiments. (B) Representative bioluminescence images of mice treated with different CAR-T cells. Colors indicate the intensity of luminescence (red, highest; blue, lowest). (C) Average radiance quantification (p/s/cm^2/sr) of the luminescence is shown. Statistical significance was calculated using Dunnett's multiple

comparisons test. (D) Body weight was monitored during treatment. (E) Survival curves of mice treated with indicated CAR-T cells. The log-rank (Mantel−Cox) test was used to calculate significance. (F) Serum cytokine levels were evaluated by ELISA 24-hours after CAR-T cells infusion. Data are plotted as the means ± SDs. *P < 0.05, **P < 0.01, ***P < 0.001, and ns: not statistically significant (⩾0.05).

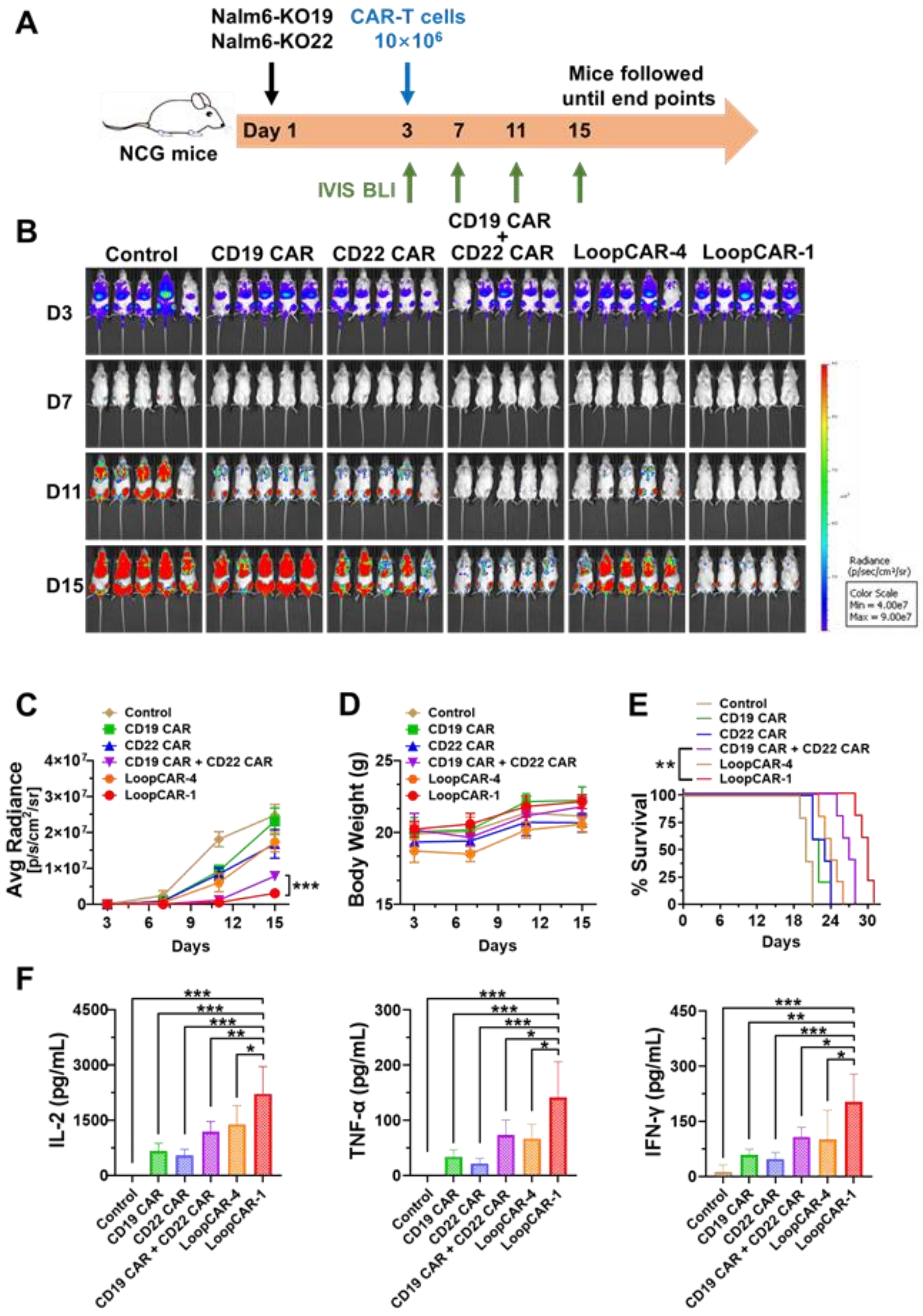

Figure S9. LoopCAR-1 improved disease control in antigen-heterogeneous tumors from two phenotypes

(A) Scheme showing the experimental design. For the heterogeneous antigen model, the mixture of luciferase-expressing Nalm6-KO19 and Nalm6-KO22 cells at 1: 1 ratio were used to maintain the immune escape model. Three days after tumor implantation, the mice (n = 5/group) were i.v. administered 10×10^6 corresponding CAR-T cells. The results were similar in two independent

experiments. (B) Representative bioluminescence images of mice treated with different CAR-T cells. Colors indicate the intensity of luminescence (red, highest; blue, lowest). (C) Average radiance quantification (p/s/cm^2/sr) of the luminescence is shown. Statistical significance was calculated using Dunnett’ s multiple comparisons test. (D) Body weight was monitored during treatment. (E) Survival curves of mice treated with indicated CAR-T cells. The log-rank (Mantel–Cox) test was used to calculate significance. (F) Serum cytokine levels were evaluated by ELISA 24-hours after CAR-T cells infusion. Data are plotted as the means ± SDs. *P < 0.05, **P < 0.01, ***P < 0.001, and ns: not statistically significant (⩾0.05).

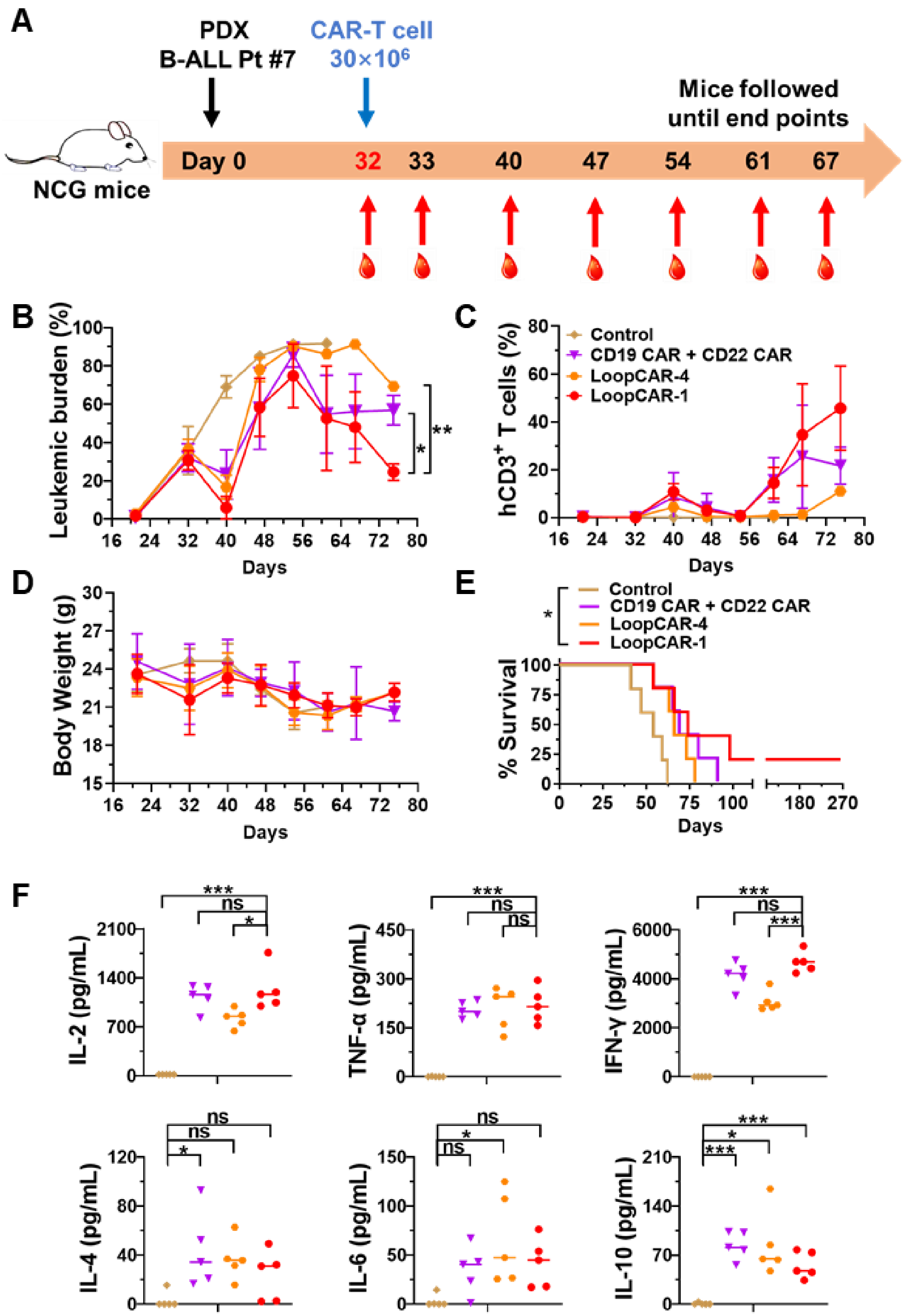

A
PDX
B-ALL Pt #7
CAR-T cell
30×10^6
Mice followed until end points
NCG mice
Day 0
32
33
40
47
54
61
67
B
Leukemic burden (%)
Days
C
hCD3+ T cells (%)
Control
CD19 CAR + CD22 CAR
LoopCAR-4
LoopCAR-1
Days
D
Body Weight (g)
Days
E
% Survival
Control
CD19 CAR + CD22 CAR
LoopCAR-4
LoopCAR-1
Days
F
IL-2 (pg/mL)
TNF-α (pg/mL)
IFN-γ (pg/mL)
IL-4 (pg/mL)
IL-6 (pg/mL)
IL-10 (pg/mL)

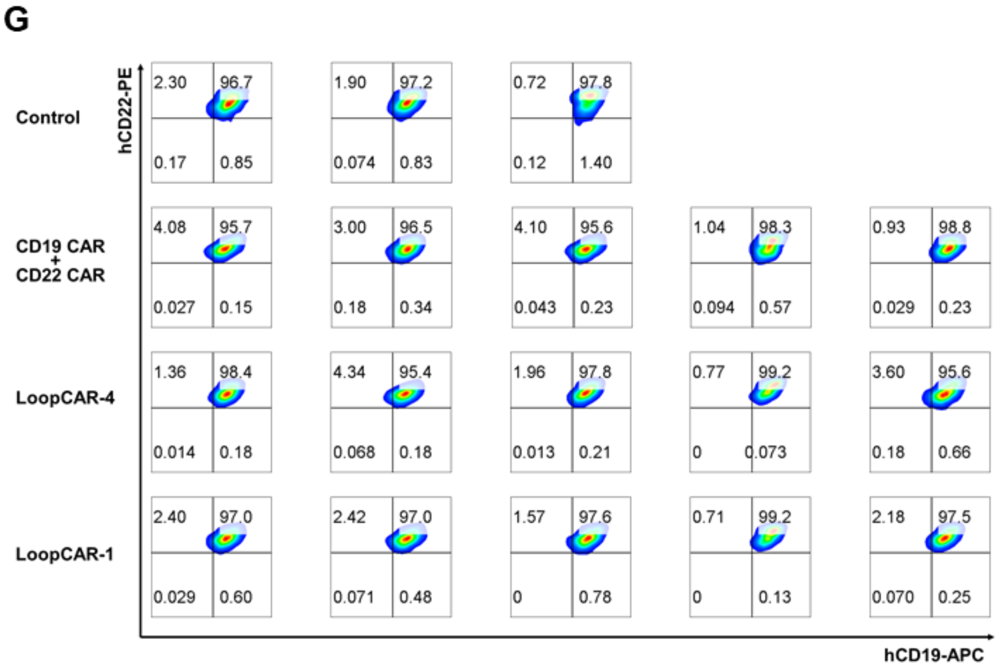

Figure S10. LoopCAR-1 controlled the disease in long-term follow-up B-ALL PDX model. (A) Scheme showing the experimental design for B-ALL PDX model. NCG mice (n = 5/group) were i.v. transplanted with 5×10^6 primary tumor cells from B-ALL Pt #7 for 32 days. Mice were randomized, and 30×10^6 corresponding CAR-T cells were i.v. injected at the indicated time points. Human B-ALL cells and human T cells in peripheral blood were analyzed by flow cytometry weekly and defined as $hCD45^+hCD10^+hCD3^-$ and $hCD45^+hCD10^-hCD3^+$, respectively. Similar results were obtained in two independent experiments. Leukemic burden in peripheral blood (B), and human T-cell persistence in peripheral blood (C) were measured over a 16-week follow-up period. (D) Body weight was monitored during treatment. (E) Survival curves of mice. The log-rank (Mantel–Cox) test was used to calculate significance. $*p < 0.05$. (F) Serum cytokine assays in the PDX model. Th1 (IL-2, IFN-γ, TNF-α) and Th2 (IL-4, IL-6, IL-10) cytokine released into the serum were evaluated 24-hours after the CAR-T cells infusion using a BD™ Cytometric Bead Array (CBA) Human Th1/Th2 Cytokine Kit. $*P < 0.05$, $**P < 0.01$, $***P < 0.001$, and ns: not statistically significant ($\geqslant 0.05$). (G) CD19 and CD22 expression levels on primary tumor cell surface were analyzed when tumor cells relapsed in CAR-treated mice.

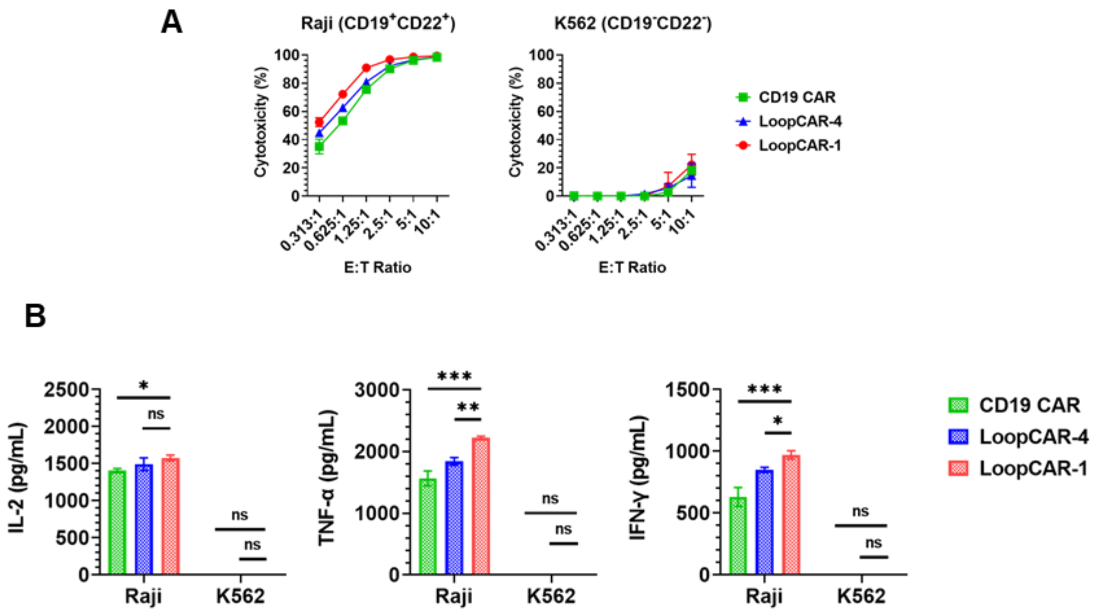

Figure S11. LoopCAR-1 demonstrated heightened sensitivity to lymphoma.

(A) Cytotoxicity comparison of CD19 CAR, LoopCAR-1 and LoopCAR-4 against Raji or K562 tumor cells after 24-hours incubation at different E: T ratios. Error bars represent means ± SDs from three independent experiments. (B) Inflammatory cytokines (IL-2, TNF-α and IFN-γ) released from CD19 CAR, LoopCAR-1 or LoopCAR-4 cocultured with Raji or K562 tumor cells for 24-hours at an E: T ratio of 1: 3 in triplicate. The data are presented as the means ± SDs from three independent experiments. And all statistical significance was calculated using the Newman−Keuls multiple comparison test: *P < 0.05, **P < 0.01, ***P < 0.001, and ns: not statistically significant (⩾0.05).

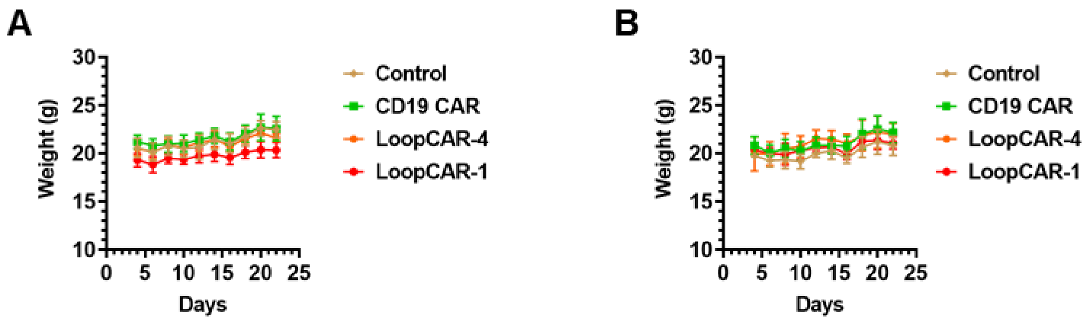

Figure S12. Body weight was monitored during treatment in the refractory lymphoma model.

After tumor inoculation, mice were administered with either (A) 3×10^6 or (B) 1×10^6 CAR-T cells per mouse.
